# Different Effects of Two Interventions Based on Cooperative Learning and PREBULLPE on Social and Emotional Competencies and Motivation in Physical Education

**DOI:** 10.1002/ejsc.70136

**Published:** 2026-02-18

**Authors:** Javier Murillo‐Moraño, Javier Raya‐González, Cristina García‐Fernández, Juan de Dios Benítez‐Sillero

**Affiliations:** ^1^ Teacher Training College “Sagrado Corazón” Ascribed to the University of Córdoba Córdoba Spain; ^2^ Research Group in Sport and Physical Education for Personal and Social Development (GIDEPSO) University of Córdoba Córdoba Spain; ^3^ Department of Specific Didactics University of Córdoba Córdoba Spain; ^4^ Department of Psychology University of Córdoba Córdoba Spain

**Keywords:** bullying, cooperative learning, motivation, physical education, social and emotional competences

## Abstract

Bullying remains one of the major challenges in education, and physical education (PE) can play a key role in its prevention while promoting students' socio‐emotional and motivational development. This study examined the effects of the *Prevention Bullying in Physical Education* (PREBULLPE) programme and cooperative learning pedagogical practice on social and emotional competencies and student motivation in PE classes. The evaluation of both educational programmes was conducted with a total of 330 primary and secondary school students (aged 11–16 years), divided into three groups: the control group (CG, *n* = 116, *M* = 13.24 and SD = 1.92), the cooperative learning experimental group (CL, *n* = 104, *M* = 12.98 and SD = 1.79), and the PREBULLPE experimental group (PB, *n* = 110, *M* = 13.92 and SD = 1.92). A quasi‐experimental design was used, with two time point measurements conducted. The Social and Emotional Competencies Questionnaire (SEC‐Q) and the Motivation Questionnaire in PE (MQPE) were used. A paired‐samples *t*‐test was applied to evaluate within‐group differences, and an analysis of covariance (ANCOVA) was performed to detect possible between‐group differences, assuming baseline values as covariates. Results showed that social and emotional competencies improved only with the PREBULLPE programme, although there were no significant differences among groups. Autonomous motivation in PE classes improved in both programmes, showing significant differences from the control group. In conclusion, PREBULLPE is effective in improving socio‐emotional competencies and autonomous motivation in PE. Cooperative learning, although not enhancing these competencies, contributes to increasing motivation. These findings highlight the need to implement specific PE programmes to prevent bullying and foster students' holistic development.

## Highlights

- The PREBULLPE programme was effective in improving both socio‐emotional competencies and motivation in physical education lessons.
- Cooperative learning mainly influenced motivational aspects but showed limited effects on socio‐emotional competencies.
- PREBULLPE incorporated specific psychosocial strategies that enhanced socio‐emotional development and bullying prevention.
- Findings highlight the potential of PREBULLPE to promote student well‐being and improve the school climate.

## 1 Introduction

The promotion of positive interpersonal relationships within the school context is a key factor in fostering coexistence and preventing school violence (Llorent et al. 2021; Ortega‐Ruiz 2015; Ortega‐Ruiz et al. 2010; Zych et al. 2021). Bullying, widely recognised as an unethical and harmful act, is defined as a repeated and intentional form of aggression in which an individual or group—holding greater physical, social or psychological power—intimidates, humiliates or harms another individual who is largely unable to defend themselves (Jiménez‐Barbero et al. 2020; Ortega Ruiz 2020).

Research on bullying behaviour has highlighted the crucial influence of both personal and contextual factors in preventing victimisation and aggression (García‐Fernández et al. 2018; Zych et al. 2021). Among these factors, social and emotional competence has been identified as essential for promoting educational success, facilitating social adjustment among peers and reducing problematic behaviours such as bullying (Camodeca et al. 2015; Romera et al. 2022). Recently, Romera et al. (2022) demonstrated the moderating role of age and gender in victimisation and aggression, indicating that girls tend to exhibit higher levels of social competence and, consequently, lower involvement in aggressive behaviours. Furthermore, preadolescence is considered a particularly relevant period for the formation and development of values and social norms adapted to the given context (Allen and Loeb 2015; Romera et al. 2022).

Scientific studies have explored social and emotional competence as a multidimensional construct, incorporating various cognitive, affective and social skills (Martín‐Criado and Casas 2019; Romera et al. 2022). In this regard, Gómez‐Ortiz et al. (2017) identified five key dimensions that define the quality of interpersonal relationships: prosociality, cognitive reappraisal, social adjustment, normative adjustment and self‐efficacy. Similarly, Zych et al. (2018) distinguished four categories related to the development of social and emotional competencies: (1) self‐awareness, which enables a better understanding of one's own emotions and their influence on thoughts and behaviours, (2) self‐management, which relates to emotional and behavioural regulation to achieve personal goals, (3) social awareness and prosocial relational skills, which are essential for understanding social contexts and ensuring normative and social adjustment and (4) responsible decision‐making, which involves considering both personal and others' interests.

In the educational context and specifically in physical education (PE), motivation towards this subject is one of the key pillars underpinning the development of social and emotional competencies (Llerandi‐Padrón and Barrios‐Palacios 2022). PE not only involves the acquisition of motor skills but also fosters social interaction and coexistence among students (Benítez‐Sillero et al. 2020). Indeed, research has demonstrated that higher motivation towards PE can enhance participation in physical and sporting activities beyond the school environment (Hagger and Chatzisarantis 2014). Furthermore, studies have shown that motivation in PE can impact health outcomes (Trigueros et al. 2019) as well as students' social and emotional well‐being (Bagøien et al. 2010).

For the study of motivation in PE, recent research by White et al. (2021) has focused on analysing it through self‐determination theory (SDT) (Ryan and Deci 2017). SDT conceptualises self‐determination along a continuum, reflecting the extent to which a behaviour is regulated by the self. Rather than merely being voluntary, self‐determined actions are those that are internally endorsed and integrated into one's sense of self. At one end of the continuum, autonomous motivations include intrinsic motivation, the highest level of self‐determination, where individuals engage in an activity due to the inherent satisfaction it provides rather than external rewards or outcomes. Following this, identified regulation occurs when an individual recognises the personal and social benefits of an action and willingly integrates it into their values and goals, even if the behaviour was initially introduced by external factors. However, intrinsic motivation and identified regulation are often confused due to their similar terminology among adolescent students (Lonsdale et al. 2011). Studies indicate that these autonomous motivations are associated with greater participation in extracurricular physical activity (Sánchez‐Oliva et al. 2020), increased involvement in sporting events (Ferriz et al. 2016) and lower levels of bullying perpetration and victimisation (Montero‐Carretero et al. 2020).

On the other hand, controlled motivations include introjected regulation (Jungert et al. 2016) and external regulation. These dimensions refer to engaging in an activity to avoid feelings of guilt or in response to internal or external psychological pressures. At the lowest level of self‐determination lies amotivation, characterised by a lack of intention to act and the absence of intrinsic or extrinsic motivational drives. In this state, individuals may feel incompetent or perceive no value in the behaviour, leading to disengagement (Ryan and Deci 2017).

According to SDT, satisfying the basic psychological needs of autonomy, competence and relatedness is essential for the development of autonomous motivation. Autonomy refers to an individual's sense of volition and self‐endorsement in their actions; competence pertains to the feeling of efficacy in performing a task; and relatedness is defined as the sense of connection, safety and integration within the social context (Ryan and Deci 2017). In the context of PE, studies suggest that individuals who satisfy these needs exhibit greater autonomous motivation and lower levels of controlled motivation (Vasconcellos et al. 2020).

### 1.1 Intervention Programs in PE: A Relevant Context for Specific Strategies

Didactic proposals in PE can contribute to enhancing social and emotional aspects, as well as motivation, due to their proven benefits (Fernandez‐Rio et al. 2017; Gil‐Madrona et al. 2019). Extensive research on social and emotional competencies in relation to bullying behaviour has highlighted the need to implement programmes focusing on these areas (Romera et al. 2022). Therefore, it appears particularly valuable to develop programmes based on the PE curriculum (Martínez‐Baena and Faus‐Boscá 2019). PE provides an ideal setting for this purpose, given its potential to foster students' holistic development. Additionally, it can help create a competitive yet supportive classroom climate that prevents bullying by prioritising social and emotional competencies (Benítez‐Sillero et al. 2020, 2021).

PE classes have the advantage of being contexts where a motivational climate is fostered, serving as scenarios where students can learn social codes that will help them interact with peers in their daily lives (Cox and Williams 2008; Méndez Giménez et al. 2013). Studies on motivation in PE have examined its role in the development of personal and social aspects such as self‐esteem, emotional and behavioural regulation, and academic performance (Blanco‐Vega et al. 2019). For example, Fernandez‐Rio et al. (2017) conducted a 16‐week intervention implementing cooperative learning pedagogical practices and observed higher levels of autonomous motivation in the experimental group. Similarly, their findings indicate that autonomy‐supportive pedagogical styles promote self‐motivation in PE, which is negatively associated with bullying behaviour (Montero‐Carretero et al. 2020).

In this regard, pedagogical models in the field of PE have characterised educational practices that focus on students, teachers, content and context (Fernandez‐Rio et al. 2018). Such models allow for the development of educational strategies and the implementation of programmes aimed at improving social relations through the cultivation of individual and group responsibility (Fernández‐Río et al. 2016).

### 1.2 Cooperative Learning and the PREBULLPE Programme

Some didactic proposals appear to be useful for fostering social and emotional competencies and motivation in PE by improving the quality of school coexistence and contributing to bullying prevention. These programmes are based on the following approaches:

#### 1.2.1 Cooperative Learning Programme

This approach is grounded in various theoretical perspectives (Buchs and Butera 2015; Slavin 2014), such as the motivational perspective, which places motivation at the centre of the teaching–learning process; group cohesion; the cognitivist approach, which promotes information processing through interactions; and developmental theories such as constructivism, which suggests that cooperation supports progress within the zones of proximal development (Erbil 2020; Vygotsky and Cole 1978). This pedagogical practice provides opportunities for students to develop social and emotional skills through active methodologies. Students work in small, structured and heterogeneous groups, aiming to promote peer interaction, positive interdependence, individual responsibility, group processing and social skills (Johnson and Johnson 2005). These principles, which underpin the implementation of cooperative learning, are consistent with three basic psychological needs (Ryan and Deci 2017). In the specific context of PE teaching, educators seek to promote students' autonomy by allowing them to make their own decisions when addressing challenges. To meet the need for competence, PE teachers implement cooperative learning strategies that ensure each student's abilities contribute to achieving the set objectives. Finally, to address the need for relatedness, this pedagogical approach fosters inclusion, ensuring that all students play a meaningful role, thereby encouraging a sense of belonging and trust (Cox et al. 2009; Fernandez‐Rio et al. 2017). The application of this approach may enhance students' perception of achievement in the PE lesson (Bores‐García et al. 2020), which has been linked to higher levels of autonomous motivation (Fernández‐Espínola et al. 2020; Liu and Lipowski 2021). Additionally, research in the field of PE suggests that this type of methodology can be efficacious in developing physical, cognitive, social and emotional skills (Casey and Goodyear 2015; Dyson et al. 2021; Dyson et al. 2022).

#### 1.2.2 Prevention Bullying in Physical Education (PREBULLPE)

This programme is designed around psychosocial strategies for bullying prevention within PE. It consists of six sessions (1 hour per session), each focusing on different aspects: understanding bullying through body expression; fostering collaboration against bullying via cooperative and noncompetitive physical challenges; promoting self‐esteem, empathy and tolerance towards diversity through group activities and imitation; developing self‐control and resilience against bullying through team games that require overcoming mistakes and team inequalities; empowering bystanders through cooperation–opposition games; and encouraging perspective‐taking by engaging in sports activities where students must chase a partner without being caught (Benítez‐Sillero et al., 2021). The programme was positively evaluated by students, possibly due to the diversity and innovative nature of its didactic proposals, with cooperative activities being among the most valued (Benítez‐Sillero et al. 2019).

As highlighted in previous research, efforts have been directed towards adapting students' social behaviour to their immediate environment, proposing educational strategies that may enhance social and emotional competencies (Romera et al. 2022). At the same time, there is a continuous research focus on developing interventions aimed at improving students' autonomous motivation, with Spain being one of the countries with a high number of studies in this field (Chen et al. 2022). However, research assessing the impact and efficacy of intervention programmes remains limited, and results appear to vary depending on the strategies, methodologies and evaluation methods used (Fernández‐Espínola et al. 2020; Gaffney et al. 2019; Liu and Lipowski 2021; Vega‐Osés and Peñalva‐Vélez 2018; Zych et al. 2015).

Therefore, the aim of this study was to examine changes associated with two intervention programmes in the field of physical education (PE) in relation to students' social and emotional competencies and motivation. Specifically, the study independently examined the effects of the cooperative learning methodology and the PREBULLPE programme. Based on previous evidence, two hypotheses were proposed: the first hypothesis stated that the cooperative learning methodology would be associated with improvements in students' social and emotional competencies as well as in their motivation towards PE lessons, while the second hypothesis proposed that the PREBULLPE programme would be associated with significant improvements in these same outcomes.

## 2 Methods

### 2.1 Study Design

A quasi‐experimental pretest/posttest design (Ato et al. 2013) with three groups was used to assess the effects of two PE interventions (i.e., cooperative learning and PREBULLPE) on motivation, responsibility and social and emotional competencies in young students. At baseline and post‐intervention phases, students completed several questionnaires regarding the aforementioned variables in a single session. The intervention programs comprised six sessions (i.e., one hour per session) and were implemented in the PE subject schedule, during the second trimester. The control group carried out its PE lessons following the regular curriculum. Although the teachers did not specify the methodology used, the content delivered included standard PE topics such as sports, physical conditioning and alternative sports. In none of the cases did the lessons implement cooperative learning or the PREBULLPE programme.

## 3 Participants

A total of 330 students, aged 11–16 years, from two public‐private schools in Córdoba participated in the study. The selected students were in the fifth and sixth years of compulsory primary education (11–12 years old) and in the first to fourth years of compulsory secondary education (13–16 years old), within a medium socio‐economic context. The groups were assigned based on school classes for convenience as follows: control group (CG, *n* = 116), cooperative learning experimental group (CL, *n* = 104) and PREBULLPE experimental group (PB, *n* = 110). The methodology used in both educational stages was the same; however, adaptations were made in the explanations according to the educational level and the class type, taking into account the specific characteristics of each class.

In one of the schools, the cooperative learning (CL) intervention was implemented in all its classes, as there was only one class per grade. In the other school, which had two classes per grade, the PREBULLPE programme was applied in one class, whereas the other served as the control group (CG). The CL intervention was conducted by a single teacher, whereas the CG was taught by two teachers—one responsible for compulsory primary education and the other for compulsory secondary education.

Due to the specificity of the PREBULLPE programme's methodology, it was considered more appropriate for it to be delivered by a researcher with experience in its implementation. Consequently, it was led by one of the researchers involved in the development of the programme, who had also been a teacher at both schools the previous year. Their extensive knowledge of the programme ensured its faithful adherence to the proposed methodology. Following the six‐session intervention, students in the experimental groups resumed classes with their regular PE teacher. Descriptive data on the students in each group are presented in Table 1.

**TABLE 1:** Descriptive data for each group (mean ± standard deviation).


Variable	CG	CL	PB
Sample (n)	116	104	110
Males (n)	61	55	53
Females (n)	55	49	57
Age (years)	13.24 ± 1.92	12.98 ± 1.79	13.12 ± 1.92

Abbreviations: CG = control group; CL = cooperative learning experimental group; PB = PREBULLPE experimental group.

### 3.1 Procedures

Between February and April, classes were assigned to the different groups: CG, CL or PB. The allocation was not random at the individual level; rather, intact classes were assigned as a whole to each study group. Before administering the questionnaires, informed consent was obtained by providing families with detailed information about the programme at the educational centre. All students were informed about the procedures, potential risks and benefits of the study, with their participation being anonymous, confidential and voluntary. Consent forms required the signature of a legal representative to authorise student participation in the questionnaires. The study was conducted in accordance with the Declaration of Helsinki and approved by the ethics committee of Córdoba (approval date: 24 January 2022). All questionnaires were administered in the classroom via computers, using Google Forms, in an anonymous and confidential manner. The time required to complete the questionnaire ranged from 20 to 30 min. The principal investigator was responsible for administering the questionnaires in both the pre‐test and posttest phases.

The intervention period consisted of six sessions (1 hour per session), conducted within the regular PE schedule. This duration was determined by the structure of the PREBULLPE programme, which is specifically designed to be implemented over six sessions. To ensure methodological consistency and comparability between interventions, the cooperative learning programme was adjusted to the same number of sessions, thereby equalising the intervention length across groups and avoiding differences attributable to intervention duration rather than to the pedagogical approach itself. Participants in the CG attended their usual PE classes, taught by their regular teacher, who maintained their standard teaching practices. The teacher confirmed that cooperative learning methodology was not applied in any of their classes, as they lacked prior training in it. After the study was completed, the teacher received training in both the cooperative learning methodology and the PREBULLPE programme.

For CL, a didactic unit consisting of challenges, games and cooperative activities based on PE motor skills was integrated into the educational curriculum (Fernández‐Río et al. 2013). Although the teacher had prior knowledge of this methodology, it was not implemented in a structured manner. To facilitate its proper application, the principal researchers of the project conducted a two‐hour training session, covering the theoretical foundations of cooperative learning, practical implementation strategies and examples of structured activities. Additionally, detailed dossiers outlining the methodology and the six planned one‐hour sessions were provided. To ensure efficacious implementation, the principal investigator maintained ongoing communication with the teacher, addressing any questions and discussing potential challenges that might arise during the sessions.

Lastly, the PB group completed the PREBULLPE programme (Benítez‐Sillero et al. 2021), which combined psychosocial content (e.g., knowledge of bullying, roles of victim and aggressor, recognition and expression of basic emotions, importance of the social group, collaborative work, self‐esteem, empathy, self‐control, resilience, discrimination, and the roles of defenders, aggressors and victims) with methodological PE strategies (e.g., cooperative games or challenges, body expression, motor storytelling, body awareness and limitation activities, motor games with symbolic roles, relay games emphasising rule adherence and competitive games adapted by modifying roles). A description of the activities for both interventions is provided in Table 2. Access to the completed educational units is available upon request, subject to authorisation from the study authors.

**TABLE 2:** Description of the PREBULLPE programme and the cooperative learning methodology over six sessions in physical education.

	PREBULLPE		Cooperative learning	
Sessions	Main goal	Physical education strategies and contents	Main goal	Physical education strategies and contents
1	Concept of bullying	Body expression is used through motor stories where the actions described by the teacher are reproduced by the students with their bodies. They play in pairs, imitating emotions according to the teacher's description and freely express themselves with music.	Cooperation: a process aimed at learning to live together in peace	The learning process of what it means to cooperate with peers is achieved through games in pairs and groups, aiming to foster trust among students. Games involving contact between students are conducted, concluding with a question about whether anyone has felt insecure during the activities
2	Collaboration against bullying	Cooperative activities are used to demonstrate the importance of teamwork, strengthening interpersonal relationships, promoting well‐being and preventing exclusion. Cooperative challenges with mats and balls are included, avoiding competition.	Satisfaction and personal well‐being through sharing and cooperating	The aim to foster greater group cohesion continues through activities such as cooperative mini tennis, where students aim to achieve the maximum number of hits without competing. Additionally, problem‐solving activities involve keeping the groups on a mat and flipping it without anyone stepping off.
3	Self‐esteem, empathy and tolerance of diversity	Collaborative activities aiming to understand oneself and others. It seeks to encourage students to empathize with others and tolerate differences. Collaborative motor challenges, such as a circuit where some individuals have simulated motor disabilities, and their peers act as assistants to help them achieve the goal.	Observation and self‐control through cooperative work	Activities begin in pairs and conclude in a group setting. The teacher presents challenges within the groups, such as shooting baskets in teams, where the combined score must surpass a target set by the teacher. The goal for students is to develop self‐control and avoid making negative comments when a teammate does not contribute points towards the final objective.
4	Self‐control and resilience against bullying	Management of issues that arise during activities, always respecting the rules and applying them to everyday situations. Activities deliberately introduce imbalances between teams and referee errors to foster the development of self‐control in students. The session concludes with a partner‐based activity aimed at mutually helping each other relax.	Fair play and well‐played games	Active participation without the possibility of failure, creating an environment that promotes trust, teamwork and collective learning, as the pressure of individual failure is eliminated, emphasizing collaboration to achieve common goals. Cooperative challenges are carried out using hoops and balls. To conclude, a general reflection is carried out followed by an evaluation and co‐evaluation questionnaire.
5	Empower the viewer against bullying	Implementation of symbolic motor games to raise awareness about discrimination situations and the role of the advocate. Cooperative–opposition games are proposed where some students hold more importance than others, emphasizing the significance of assuming each role.	Positive interdependence and individual responsibility	Cooperative challenge circuit in groups, where students must demonstrate commitment and interpersonal skills in each challenge, as everyone are essential to meet the goal set by the teacher. For instance, in one of the challenges, the entire group must pass under a rope without touching it with their hands or arms; if anyone in the group does so, they must start over from the beginning.
6	Put yourself in the victim's place	Experimenting with the roles of victims and aggressors through motor play involving symbolic roles. Activities include scenarios like pursuing a protagonist and crossing a line of companions without being caught.	Reinforcing the cooperative nature of the proposed activities	Recap of all the knowledge gained in previous sessions. Various cooperative challenges are presented for students to choose which ones to tackle. An example is ‘the wall', where the goal is for all group members to cross a vertically arranged high‐jump mat ensuring it does not fall. Finally, there is a group reflection proposal on the experiences and an individual sociometric test.

*Note:* Adapted and modified from Benítez‐Sillero et al. (2021), including the cooperative learning methodology in physical education.

### 3.2 Instruments

#### 3.2.1 Social and Emotional Competencies Questionnaire (SEC‐Q)

The questionnaire validated by Zych et al. (2018) was applied to assess the social and emotional competencies of the students. Through a Likert‐type scale, participants showed their agreement with the 16 statements purposed [from one (totally disagree) to five (totally agree)]. For example, “*I usually know how to help people in need, or I get along with my classmates*”. From these, four variables are obtained—self‐awareness (SelfAw), self‐management and motivation (SelfMa), social behaviour and prosocial behaviour (SocBe) and decision‐making (DecMa). For this questionnaire, the internal consistency was as follows: Ω self‐awareness *T*1 = 0.66, Ω self‐awareness *T*2 = 0.79, Ω self‐management and motivation *T*1 = 0.70, Ω self‐management and motivation *T*2 = 0.75, Ω social behaviour and prosocial behaviour *T*1 = 0.74, Ω social behaviour and prosocial behaviour *T*2 = 0.72, Ω decision‐making *T*1 = 0.66 and Ω decision‐making *T*2 = 0.70.

#### 3.2.2 Motivation Questionnaire in PE (MQPE)

This questionnaire was used to assess the variables related to motivation [that is, intrinsic motivation (IntMot), identified regulation (IdReg), introjected regulation (IntReg), external regulation (ExtReg) and demotivation (Demot)], which was previously validated by Sánchez‐Oliva et al. (Sánchez‐Oliva et al. 2012). This questionnaire was composed of 20 statements with Likert‐type response options, from one (totally disagree) to five (totally agree). For example, “*I participate in PE lessons… because this subject is pleasant and interesting to me, or because I value the benefits that this subject can have to develop me as a person*”. For this questionnaire, the internal consistency was as follows: Ω intrinsic motivation *T*1 = 0.76, Ω intrinsic motivation *T*2 = 0.82, Ω identified regulation *T*1 = 0.78, Ω identified regulation *T*2 = 0.81, Ω introjected regulation *T*1 = 0.66, Ω introjected regulation *T*2 = 0.75, Ω external regulation *T*1 = 0.78, Ω external regulation *T*2 = 0.82, Ω demotivation *T*1 = 0.70 and Ω demotivation *T*2 = 0.82.

Based on the variables related to the motivation, the self‐determination index (SelfDet) was calculated with the following formula: SelfDet = (2 x IntMot + IdReg) – [(IntReg + ExtReg)/2 + 2 x Demot] (R. Vallerand and Rousseau 2001). A high value in this index will mean a high self‐determination. Despite criticisms of the Self‐Determination Index (SelfDet) for not accounting for the contextual influence on the quality of motivation (Martín‐Albo et al. 2014; R. J. Vallerand et al. 2008) its use remains relevant in studies aiming to measure the degree of self‐determination in motivation (Montero‐Carretero et al. 2020; Sanchez‐De Miguel et al. 2023). In this study, the SelfDet was employed due to its ability to provide an overall assessment of motivational quality, enabling comparisons between groups. However, it was complemented by an analysis of the different types of motivation to allow for a more detailed interpretation of the results.

### 3.3 Statistical Analysis

Descriptive statistics are presented as mean ± standard deviation (SD) and standard error (SE). For the calculation of internal reliability, McDonald's omega (Ω) was considered. The Shapiro–Wilk and Levene tests were used to assess the normality of the distribution and the homogeneity of variances, respectively. A repeated measures analysis of covariance (rm‐ANCOVA) was conducted to evaluate both within‐group and between‐group differences. For the within‐group effects, the model adjusted for the covariates of gender and age, whereas for the between‐group differences, the baseline measure was also included as a covariate to control for potential pretest differences between groups. The analysis was carried out using the estimated marginal means to interpret the adjusted effects, and Bonferroni correction was applied to reduce Type I error. Effect sizes (ES) were calculated using Cohen's d and interpreted as follows: < 0.2, trivial; 0.20–0.49, small; 0.50–0.80, moderate; and > 0.80, large (Cohen 1988). Effect sizes (partial eta‐squared ηp2) for practical significance of ANCOVA were considered with the following interpretation: < 0.02, small; 0.02–0.26, medium; and > 0.26, large (Pierce et al. 2004). The data analysis was performed using SPSS version 29, with statistical significance set at *p* < 0.05.

## 4 Results

Table 3 shows the changes in social and emotional competencies after the experimental period. Nonsignificant differences (*p* < 0.05) were observed in CG and CL for any variable. In PB, significant differences were found in SelfAw (*p* = 0.041; ES = 0.168) and SocBe (*p* = 0.032; ES = 0.191). Between‐group analysis revealed no significant differences for any variable, nor was there any interaction among group, time and gender.

**TABLE 3:** Changes in social and emotional competencies after the experimental period (estimated marginal means with age and gender control).

	CG (*n* = 116)					CL (*n* = 104)					PB (*n* = 110)				
Variable	Baseline Mean ± SE	Post‐intervention Mean ± SE	Δ (%)	*p*	ES	Baseline Mean ± SE	Post‐intervention Mean ± SE	Δ (%)	*p*	ES	Baseline Mean ± SE	Post‐intervention Mean ± SE	Δ (%)	*p*	ES
SelfAw	14.67 ± 0.27	15.00 ± 0.30	2.25	0.306	0.107	14.90 ± 0.28	15.34 ± 0.32	2.95	0.190	0.144	15.06 ± 0.28	15.58 ± 0.31	3.45	**0.041**	**0.168**
SelfMa	11.63 ± 0.24	11.72 ± 0.24	0.77	0.657	0.035	12.51 ± 0.25	12.64 ± 0.25	1.04	0.563	0.051	12.34 ± 0.24	12.65 ± 0.24	2.41	0.166	0.123
SocBe	23.87 ± 0.33	24.09 ± 0.31	0.92	0.459	0.064	23.83 ± 0.35	24.01 ± 0.32	0.76	0.561	0.053	24.06 ± 0.34	24.72 ± 0.32	2.74	**0.032**	**0.191**
DecMa	9.73 ± 0.24	9.82 ± 0.25	0.92	0.718	0.034	10.54 ± 2.62	10.39 ± 0.27	−1.42	0.570	0.008	10.65 ± 0.25	10.55 ± 0.26	−0.94	0.808	0.037

*Note:* The bold values indicate statistical significance (*p* < 0.05).

Abbreviations: CG = control group; CL = cooperative learning experimental group; DecMa = decision‐making; ES = effect size; *p* = level of significance; PB = PREBULLPE experimental group; SE = standard error; SelfAw = self‐awareness; SelfMa = self‐management and motivation; SocBe = social behaviour and prosocial behaviour; Δ (%) = percentage of change between pre‐ and post‐intervention.

Changes in motivation‐related variables after the experimental period are shown in Table 4. Nonsignificant differences (*p* < 0.05) were observed in CG for any variable. Regarding to CL, a significant decrement in IntReg (*p* = 0.008; ES = 0.229) and a significant increase in ExtReg (*p* = 0.008; ES = 0.225) were found. Finally, significant increases for IntMot (*p* = 0.014; ES = 0.247), IdeReg (*p* = 0.031; ES = 0.206), ExtReg (*p* = 0.003; ES = 0.235) and SelfDet (*p* = 0.016; ES = 0.243) were observed in PB. No significant interaction among group, time and gender was found for any of the variables.

**TABLE 4:** Changes in motivation‐related variables and in personal responsibility after the experimental period (estimated marginal means with age and gender control).

	CG (*n* = 116)					CL (*n* = 104)					PB (*n* = 110)				
Variable	Baseline Mean ± SE	Post‐intervention Mean ± SE	Δ (%)	*p*	ES	Baseline Mean ± SE	Post‐intervention Mean ± SE	Δ (%)	*p*	ES	Baseline Mean ± SE	Post‐intervention Mean ± SE	Δ (%)	*p*	ES
IntMot	4.15 ± 0.07	4.09 ± 0.06	−1.45	0.387	0.088	4.26 ± 0.07	4.38 ± 0.07	2.82	0.094	0.174	4.17 ± 0.07	4.34 ± 0.06	4.08	**0.014**	**0.247**
IdReg	3.82 ± 0.08	3.72 ± 0.08	−2.62	0.192	0.107	4.00 ± 0.08	4.05 ± 0.08	1.25	0.622	0.061	4.00 ± 0.08	4.17 ± 0.08	4.25	**0.031**	**0.206**
IntReg	3.14 ± 0.09	3.06 ± 0.09	−2.55	0.350	0.121	3.30 ± 0.09	3.09 ± 0.09	−6.36	**0.008**	**0.229**	3.19 ± 0.09	3.33 ± 0.09	4.39	0.070	0.148
ExtReg	2.98 ± 0.10	2.89 ± 0.10	−3.02	0.352	0.077	2.86 ± 0.11	3.10 ± 0.10	8.39	**0.008**	**0.225**	2.96 ± 0.10	3.21 ± 0.10	8.45	**0.003**	**0.235**
Demot	1.50 ± 0.06	1.59 ± 0.07	6.00	0.165	0.123	1.45 ± 0.07	1.40 ± 0.08	−3.45	0.448	0.068	1.35 ± 0.06	1.43 ± 0.08	5.93	0.275	0.109
SelfDet	10.81 ± 0.17	10.67 ± 0.17	−1.30	0.579	0.079	11.21 ± 0.18	11.45 ± 0.17	2.14	0.156	0.135	11.00 ± 0.17	11.43 ± 0.17	3.91	**0.016**	**0.243**

*Note:* The bold values indicate statistical significance (*p* < 0.05).

Abbreviations: CG = control group; CL = cooperative learning experimental group; Demot = demotivation; ES = effect size; ExtReg = external regulation; IdReg = identified regulation; IntMot = intrinsic motivation; IntReg = introjected regulation; *p* = level of significance; PB = PREBULLPE experimental group; SE = standard error; SelfDet = self‐determination index; Δ (%) = percentage of change between pre‐ and post‐intervention.

Repeated measures’ analysis with covariates (ANCOVA) revealed a significant interaction effect between time and the control/experimental group in various dimensions of motivation. Regarding IntMot (*F* = 6.053, *p* = 0.002 and *η*p^2^ = 0.036), both CL and PB groups exhibited superior results to CG (*p* = 0.01 and *p* = 0.008, respectively). For IdeReg (*F* = 7.057, *p* = 0.001 and *η*p^2^ = 0.042), PB showed significantly higher scores than CG (*p* < 0.001). In IntReg (*F* = 5.359, *p* = 0.005 and *η*p^2^ = 0.032), PB outperformed both CL (*p* = 0.007) and CG (*p* = 0.039). For ExtReg (*F* = 5.610, *p* = 0.004 and *η*p^2^ = 0.034), PB achieved higher scores than CG (*p* = 0.006), while CL also outperformed CG (*p* = 0.031). Finally, in SelfDet (*F* = 6.048, *p* = 0.003 and *η*p^2^ = 0.036), both PB and CL obtained higher scores than CG (*p* = 0.005 and *p* = 0.018, respectively). Additionally, no significant interactions were found among group, time and gender for any of the variables; therefore, post hoc analyses were not conducted.

## 5 Discussion

The aim of this study was to analyse changes associated with two intervention programmes implemented during physical education (PE) lessons on social and emotional competencies and motivation towards the subject. Based on this, two hypotheses were proposed: (1) the cooperative learning programme would be associated with improvements in social and emotional competencies and motivation in PE lessons and (2) the PREBULLPE programme would promote the development of social and emotional competencies as well as motivation.

The results suggest that although both programmes produced changes in motivation variables, only the PREBULLPE programme showed positive changes in increasing both social and emotional competencies and motivation. This finding partially supports the first hypothesis, as cooperative learning did not produce significant improvements in socio‐emotional competencies but did have an impact on some motivational aspects. In contrast, it provides support for the second hypothesis, reinforcing the potential usefulness of PREBULLPE in preventing social phenomena such as bullying because previous studies have identified these factors as protective (Romera et al. 2022).

Specifically, regarding the primary aim of the study, the results indicate that social and emotional competencies did not improve in the control group. Similarly, the cooperative learning programme did not show significant changes in any of the assessed variables, in contrast to previous research suggesting that this methodology enhances social and emotional skills (Dyson et al. 2021; Dyson et al. 2022). This discrepancy could be attributed to the short duration of the intervention, which may not have provided sufficient time for noticeable changes among students. In contrast, the PREBULLPE programme was associated with positive outcomes, as it applies psychosocial strategies designed to enhance these aspects and prevent bullying behaviours, in line with prior studies supporting its efficacy (Benítez‐Sillero et al. 2021). A possible explanation for these differing outcomes is that while cooperative learning primarily emphasises collaboration and teamwork (Fernández‐Río et al. 2013), the PREBULLPE programme directly targets the development of emotional and social skills. Another factor that may have contributed to the difference is that the PREBULLPE programme was implemented by a specialised researcher, ensuring a more consistent and faithful application of the intervention, which likely reinforced its efficacy.

However, the study's findings indicate that improvements in social and emotional competencies were limited to two specific areas: self‐awareness and social awareness, as well as prosocial relational skills in the PB group. No significant differences were observed compared to the control group. These elements contribute to a better understanding of one's own emotions and those of others, as well as a greater awareness of the social context, which facilitates improved peer interactions. These factors are crucial in preventing both victimisation and aggression, as highlighted in previous research (Romera et al. 2022). These findings provide valuable insights for selecting the most appropriate PE programmes or even integrating multiple approaches to enhance cognitive, affective and social development (Shen and Shao 2022).

Regarding motivation, the results suggest that both the cooperative learning intervention and the PREBULLPE programme contributed to increased intrinsic motivation and the self‐determination index in PE lessons compared to the control group. These findings partially support the first hypothesis and confirm the second, and they align with recent meta‐analyses in the field, which indicate that cooperative learning in PE can enhance students' intrinsic motivation (Fernández‐Espínola et al. 2020; Liu and Lipowski 2021). However, it is important to note that previous studies have not specifically examined the self‐determination index, limiting direct comparisons regarding this variable.

One potential explanation for the increase in intrinsic motivation in the PREBULLPE programme is the variety of content used, such as motor storytelling and motor games with symbolic roles—strategies that are rarely incorporated into PE classes but have been found engaging for students in previous research (Benítez‐Sillero et al. 2019). Additionally, the PREBULLPE programme, like the cooperative learning group, utilised cooperative challenges with a noncompetitive focus. Activities that minimise competition while fostering social interaction and positive interdependence tend to enhance students' sense of achievement in PE classes (Bores‐García et al. 2020). This, in turn, can lead to greater satisfaction of basic psychological needs (Cox et al. 2009; Ryan and Deci 2017), which is known to improve intrinsic motivation (Liu and Lipowski 2021).

Moreover, the PREBULLPE group exhibited an increase in identified regulation compared to the control group. However, it is often difficult to distinguish this variable from intrinsic motivation in adolescents (Lonsdale et al. 2011). Given the characteristics of the participants in this study, identified regulation can be generally considered a self‐determined form of motivation, similar to intrinsic motivation. The increase in both types of self‐determined motivation is a positive outcome for student well‐being (Trigueros et al. 2019), as it has been linked to higher engagement in extracurricular physical activity (Sánchez‐Oliva et al. 2020), greater participation in sporting events (Ferriz et al. 2016) and reduced bullying behaviours (Montero‐Carretero et al. 2020).

However, an unintended outcome was the increase in introjected regulation within the PREBULLPE programme compared to both the control and cooperative learning groups. This is undesirable, as the interventions aimed to foster autonomous motivation rather than behaviours driven by internal group pressures. Nonetheless, introjected regulation is rarely analysed in depth due to its complex distinction from more controlled forms of motivation (Jungert et al. 2016). Additionally, external regulation increased in both experimental groups compared to the control group, contrary to findings in previous cooperative learning studies (Fernandez‐Rio et al. 2017). This may be explained by students' awareness that they were participating in a specific intervention, leading them to focus on performing well in the programme rather than engaging with the activities for their own intrinsic enjoyment (Friedman et al. 2015).

Despite these findings, several limitations should be considered when interpreting the results of this study. These limitations are primarily related to the reliance on self‐reported data and the short duration of the intervention, which makes it challenging to control for certain variables. Although there was a balance in the number of participants between the quasi‐experimental groups and the control group, the sample was not diverse, as it was drawn from a limited number of schools. Regarding the control groups, it was not possible to have a separate control group for each school, as one of the schools only had one class per grade. Additionally, in the case of compulsory primary education, only students from the fifth and sixth years were included, whereas all grades of compulsory secondary education participated. This may limit the generalizability of the results to the entire primary education stage.

Another limitation concerns the implementation of the PREBULLPE programme by a different teacher from the students' regular PE teacher. This factor, rather than the PB programme itself, may have contributed to the observed significant effects. The presence of a new instructor could have influenced students' engagement and motivation, leading to a bias in the findings.

For future research, longer intervention programmes should be conducted to produce more lasting effects, as indicated by previous studies. Extending the duration of the intervention could also mitigate the initial novelty effect, potentially reducing external and introjected regulation, as students become more accustomed to the activities over time. Furthermore, to avoid biases associated with instructor changes, future studies should ensure that interventions are delivered by the students' regular PE teachers, who should receive prior training in the programme. This would provide a more realistic assessment of the programme's impact on all dimensions of motivation.

Additionally, further research is needed to explore the interplay between social and emotional competencies and bullying behaviours, including emerging forms such as cyberbullying. A more detailed examination of these interactions could help design targeted interventions tailored to specific student populations, improving the effectiveness of bullying prevention strategies.

## 6 Conclusions

This research provides new insights into the efficacy of programmes aimed at improving social and emotional competencies in children and adolescents, highlighting the potential of PE in their development. However, it was observed that some programmes are more efficacious than others depending on the methodological strategies used. The pedagogical practice of cooperative learning, with a limited number of sessions, may not generate a significant impact on improving socio‐emotional competencies. In this regard, the PREBULLPE programme may be superior in improving these competencies, although more studies are needed to confirm these findings. On the other hand, the PREBULLPE programme and the pedagogical practice of cooperative learning in PE proved efficacy in improving autonomous motivation, intrinsic motivation and introjected regulation. However, an undesired effect was observed related to the increase in introjected and external regulation, although these dimensions should be analysed with caution due to the methodology used in the study.

It is important to consider that the results of the study may have been influenced by cultural or contextual factors specific to Spain. For example, social norms and educational structures can vary significantly from one country to another, which could affect the efficacy of the interventions. In addition, the shorter duration of the intervention may have limited the magnitude of the observed effects. Therefore, future research should consider longer and longitudinal study designs in order to examine the stability and sustainability of changes in social, emotional and motivational variables over time.

### 6.1 Practical Implications and Recommendations for Educational Policymakers

The educational implication of this study highlights the importance of improving interpersonal relationships and the classroom climate, which are key elements in the prevention of social issues such as bullying and cyberbullying. It also points out that different intervention programmes produce varied results, making it essential to adapt PE programmes based on the specific objectives pursued. This flexibility facilitates decision‐making when implementing intervention strategies.

For the efficacious implementation of the PREBULLPE, it is crucial to offer teachers specific training that enables them to integrate emotional and social competencies within PE classes. This training should include practical workshops on managing group dynamics and conflict resolution strategies and creating an inclusive and empathetic environment. Furthermore, professional development programmes should be provided, including practical training on the use of methodologies such as PREBULLPE and psychosocial strategies to address bullying situations in PE.

The study also suggests that hybridizing methodological approaches can maximize the positive effects of each programme, optimizing the benefits when apply separately. This opens new possibilities for developing more efficacious PE programmes that promote both social and emotional development, fostering a more inclusive and supportive environment for students.

Finally, at the educational policy level, it is essential that policymakers recognize the importance of socio‐emotional education within the official PE curriculum. This could be reflected in policies that support the continuous training of teachers in emotional competencies and the inclusion of social development activities within the official PE programmes. At the district level, guidelines should be established to allow for the consistent implementation of programmes such as PREBULLPE, facilitating the necessary resources and training for teachers.

## Funding

The authors have nothing to report.

## Conflicts of Interest

The authors declare no conflicts of interest.
